# Granulysin, a novel marker for extranodal NK/T cell lymphoma, nasal type

**DOI:** 10.1007/s00428-018-2434-x

**Published:** 2018-08-27

**Authors:** Giuseppe Lo Bello, Ayse U. Akarca, Maria Raffaella Ambrosio, Claudio Agostinelli, Hernan Molina-Kirsch, Alan Ramsay, Manuel Rodriguez-Justo, Matt Pugh, Shuchun Zhao, Monique DeLisser, Elena Sabattini, Stefan Dojcinov, Stefano A. Pileri, Yasodha Natkunam, Lorenzo Leoncini, Teresa Marafioti

**Affiliations:** 1 Department of Medical Biotechnologies, Section of Pathology, University of Siena, 53100 Siena, Italy; 2 Department of Histopathology, University College London, University College Hospital, Rockefeller Building, UCL Site, 21 University Street, London, WC1E 6JJ UK; 3 Haematopathology Unit, Department of Experimental Diagnostic and Specialty Medicine, S. Orsola-Malpighi Hospital, University of Bologna, 40138 Bologna, Italy; 4 Department of Pathology, San Juan General Hospital, 10303 Guatemala City, Guatemala; 5 Department of Cellular Pathology, University College Hospital London, London, WC1E 6JJ UK; 6 Department of Pathology, University Hospital of Wales, Cardiff, CF14 4XW UK; 7 Department of Pathology, Stanford University School of Medicine, Stanford, CA 94305-5324 USA; 8 University of Bologna, University School of Medicine, 40138 Bologna, Italy; 9 Italy and Unit of Haematopathology, European Institute of Oncology, IEO, Milan, Italy

**Keywords:** T cell lymphoma, Immunohistochemistry, Granulysin, NK-T cells, Phenotype

## Abstract

Granulysin is a cytolytic protein expressed in cytotoxic T and natural killer (NK) cells. Abnormal serum levels of granulysin in lymphomas with NK and cytotoxic phenotype have been shown to correlate with tumour progression. In this study, we investigated the expression pattern of granulysin in routine sections of normal and reactive lymphoid tissues as well as in a large series of lymphomas. In normal tissues, granulysin labelled a small population of cells that double immunostaining revealed to belong to the pool of cytotoxic T/NK cells. Among lymphoid neoplasms, the highest expression of granulysin (71%) was found in extranodal NK/T cell lymphomas of nasal type (ENKTL). To note is that 29% of ENKTLs, which were negative for one or more of classical cytotoxic markers strongly expressed granulysin. Furthermore, expression of granulysin was observed in rare cases of T cell lymphomas with a cytotoxic phenotype (i.e. ALK-negative anaplastic large cell lymphoma (26%), enteropathy-associated T cell lymphoma (12%) and peripheral T cell lymphoma, NOS (4%)). None of the investigated non-Hodgkin B cell lymphomas, Hodgkin lymphoma and plasma cell myeloma were granulysin positive. The results suggest granulysin as a novel marker for a subset of cytotoxic NK cell derived malignancies and its usefulness is highlighted in those ENKTLs that lack expression of other cytotoxic markers but retain granulysin expression.

## Introduction

Granulysin is a sposin-like lipid-binding protein that was originally described in 1998 as a human antimicrobial peptide with a broad activity against intracellular pathogens [1]. The protein was initially identified as a cytolytic and pro-inflammatory molecule expressed by natural killer (NK) cells and activated cytotoxic T lymphocytes (CTLs) [2, 3]. It is localised within granules and associated with other pore-forming proteins like perforin and granzyme [1, 4].

Several studies have shown the clinical relevance of granulysin in a number of physiologic conditions including reproductive biology, innate immunity, dendritic cell chemotaxis and activation as well as in infectious diseases and in cancers [5–7]. It has been shown that serum levels of granulysin in patients with mature NK neoplasms were abnormal and heterogeneously expressed [8]. The findings led to suggest granulysin as a potential diagnostic and prognostic marker for these lymphoma types [8].

Mature NK/T cell lymphomas are distinct clinicopathological entities and include extranodal NK/T cell lymphoma, nasal type (ENKTL), aggressive NK-cell leukaemia (ANKL) and chronic lymphoproliferative disorders of NK cells (CLPD-NK) [9]. ENKTL predominantly occurs in Asian and Central/South American populations, shows frequent involvement of extra-nodal sites [9–12] though nodal localizations are described and it is associated with Epstein-Barr virus (EBV) infection [10]. The neoplastic cells show variable expression of NK and cytotoxic T cell markers and molecular studies demonstrated T cell receptor (TCR) gene rearrangements only in a minority of cases [9].

The diagnosis of ENKTL is often challenging due to the variable morphologic and immunophenotypic features [13–15] overlapping sometimes to those of subsets of T cell lymphomas with cytotoxic phenotype and pathologists need additional and robust markers to achieve precision diagnosis. Identification of a novel marker that can help in this diagnostic setting is of interest.

In the present investigation, we carried out an immunohistochemical analysis of granulysin, in normal and neoplastic lymphoid tissues, with the aim to assess its potential utility to refine the diagnosis of NK/T cell lymphoma subtypes.

## Materials and methods

### Patients and tissue samples

Formalin-fixed paraffin-embedded (FFPE) tissue blocks were retrieved from the files of the Departments of Histopathology, University College Hospital, London, UK; Pathology, Stanford University School of Medicine, CA, USA; Pathology, University Hospital of Wales, Cardiff, UK; and from the Units of Pathology, University Hospital Siena and of Haematopathology, S. Orsola Malpighi Hospital, University of Bologna, Italy. In addition, sections of tissue microarrays (TMAs) constructed from FFPE tissue samples of T cell lymphoma subtypes, as reported previously [16], were also included. The tissue-microarrays were generated upon selection of representative, tumour-rich areas by two expert haematopathologists (YN and HM-K) and were retrieved from the files of the Departments of Pathology, Stanford University School of Medicine, Stanford, USA, and San Juan General Hospital Guatemala City, Guatemala.

The samples included in this investigation consisted of normal lymphoid tissues comprising thymus (*n* = 1), tonsils (*n* = 4), lymph node (*n* = 1), spleen (*n* = 1), two lymph nodes showing reactive changes specific of EBV-associated lymphadenitis (i.e. infectious mononucleosis), five lymph nodes with the diagnosis of chronic active EBV infection (CAEBV), and a large series of non-Hodgkin and Hodgkin lymphomas (Table 1).

**Table 1:** Expression of granulysin in haematological neoplasms

Lymphoma/leukaemia entity		*n*/*N* (%)
B cell lymphoma	Chronic lymphocytic leukaemia	0/10
	Mantle cell lymphoma	0/10
	Follicular lymphoma	0/10
	Diffuse large B cell lymphoma	0/10
	T-cell/histiocyte-rich large B cell lymphoma	0/7
	Burkitt lymphoma	0/9
	Hairy cell leukaemia	0/7
	Chronic active EBV infection (CAEBV), B cell type	0/1
T/NK cell lymphoma	Extranodal NK/T cell lymphoma, nasal type	61/86 (71%)
	ALK-negative anaplastic large cell lymphoma (ALK-ALCL)	4/15 (26%)
	Enteropathy-associated T-cell lymphoma (EATL)	1/8 (12%)
	Peripheral T-cell lymphoma, NOS (PTCL, NOS)	2/50 (4%)
	T-cell large granular lymphocytic leukaemia	0/8
	Angioimmunoblastic T-cell lymphoma	0/24
	ALK-positive anaplastic large cell lymphoma (ALK+ALCL)	0/5
	Hepatosplenic T-cell lymphoma	0/8
	T-lymphoblastic leukaemia (T-ALL)	0/6
	Adult T cell leukaemia/lymphoma (ATLL)	0/6
	CAEBV, T cell type	0/4
Hodgkin lymphoma	Classical	0/20
	Lymphocyte predominant	0/29
Plasma cells neoplasms	Myeloma	0/10

*n* number of cases positive for granulysin, *N* total number of cases, *%* percentage of positive cases, *NOS* not otherwise specified

Primary diagnosis was performed at each Institution by expert haematopathologists according to the criteria of the updated World Health Organization classification of Tumors of Haematopoietic and Lymphoid Tissues [9].

### IHC and antibodies

Immunohistochemistry (IHC) was carried out on a Bond-III Autostainer (Leica Microsystems, Newcastle upon Tyne, UK) as previously described [17]. The following mouse monoclonal antibodies raised against fixation-resistant epitopes were applied: anti-granulysin (clone F-9, dilution 1:300, Santa Cruz, Santa Cruz Biotechnology, Inc., TX, USA), anti-CD3 (clone LN10, dilution 1:100, Leica Microsystems Ltd., Newcastle-upon-Tyne, UK), anti-CD5 (clone 4C7; dilution 1:100, Leica Microsystems Ltd., Newcastle-upon-Tyne, UK), anti-CD2 (clone 11F11, dilution RTU, Leica Microsystems Ltd., Newcastle-upon-Tyne, UK), anti-CD7 (clone LP15, dilution RTU, Leica Microsystems Ltd., Newcastle-upon-Tyne, UK), anti-CD4 (clone 4B12, dilution RTU, Leica Microsystems Ltd., Newcastle-upon-Tyne, UK), anti-CD8 (clone 4B11, dilution RTU, Leica Microsystems Ltd., Newcastle-upon-Tyne, UK), anti-CD30 (clone JCM182, dilution RTU, Leica Microsystems Ltd., Newcastle-upon-Tyne, UK), anti-CD43 (clone MT1, dilution RTU, Leica Microsystems Ltd., Newcastle-upon-Tyne, UK), anti-CD56 (clone CD564, dilution RTU, Leica Microsystems Ltd., Newcastle-upon-Tyne, UK), anti-granzyme B (clone 11F1, dilution RTU, Leica Microsystems Ltd., Newcastle-upon-Tyne, UK), anti-TIA-1 (clone 2G9A10F5, dilution 1:150, Beckman Coulter Ltd., High Wycombe, UK), anti-perforin (clone 5B10, dilution 1:30, Leica Microsystems Ltd., Newcastle-upon-Tyne, UK).

To investigate the expression pattern of granulysin in lymphoid cell subsets, sections of reactive tonsil and lymph node as well as lymph node of patients with infectious mononucleosis were analysed to double immunostaining using a protocol described elsewhere [18]. The antibodies panels consisted of granulysin in combination with one of the following molecules: CD4, CD8, CD56 and granzyme B.

In ENKTL cases staining for CD56, granzyme B, TIA1 and perforin was assessed as percentage of positive tumour cells over the total number of cells.

The review and scoring of the staining was performed by expert haematopathologists (GLB, MRA, LL, YN and TM) who were blinded to each other and to the clinical history and original diagnosis.

The score to assess the percentage of positive cells over the number of all cells was set as it follows: 1 (10–25% positive cells), 2 (25–75% positive cells) and 3 (> 75% positive cells) [19].

Five different fields (at least 100 cells/field) were evaluated at × 200 magnification. Cases with discrepant scoring were reviewed jointly by two out of the four haematopathologists to reach a final consensus.

### Detection of EBV

In situ hybridization for detection of the EBV-encoded small RNAs (EBER) was performed using the INFORM EBER Probe (Ventana/ROCHE, Tucson, AZ, US). The technique was carried out in the BenchMark Ultra (Ventana/ROCHE) platform according to the manufacturer’s instructions, as previously described [20].

### Statistical analysis

Chi-square test (99% CI) and GraphPad Software (La Jolla, CA, USA) were used to calculate the *p* value in the statistical analysis.

## Results

### Granulysin expression in non-neoplastic lymphoid tissues

**Fig. 1:**
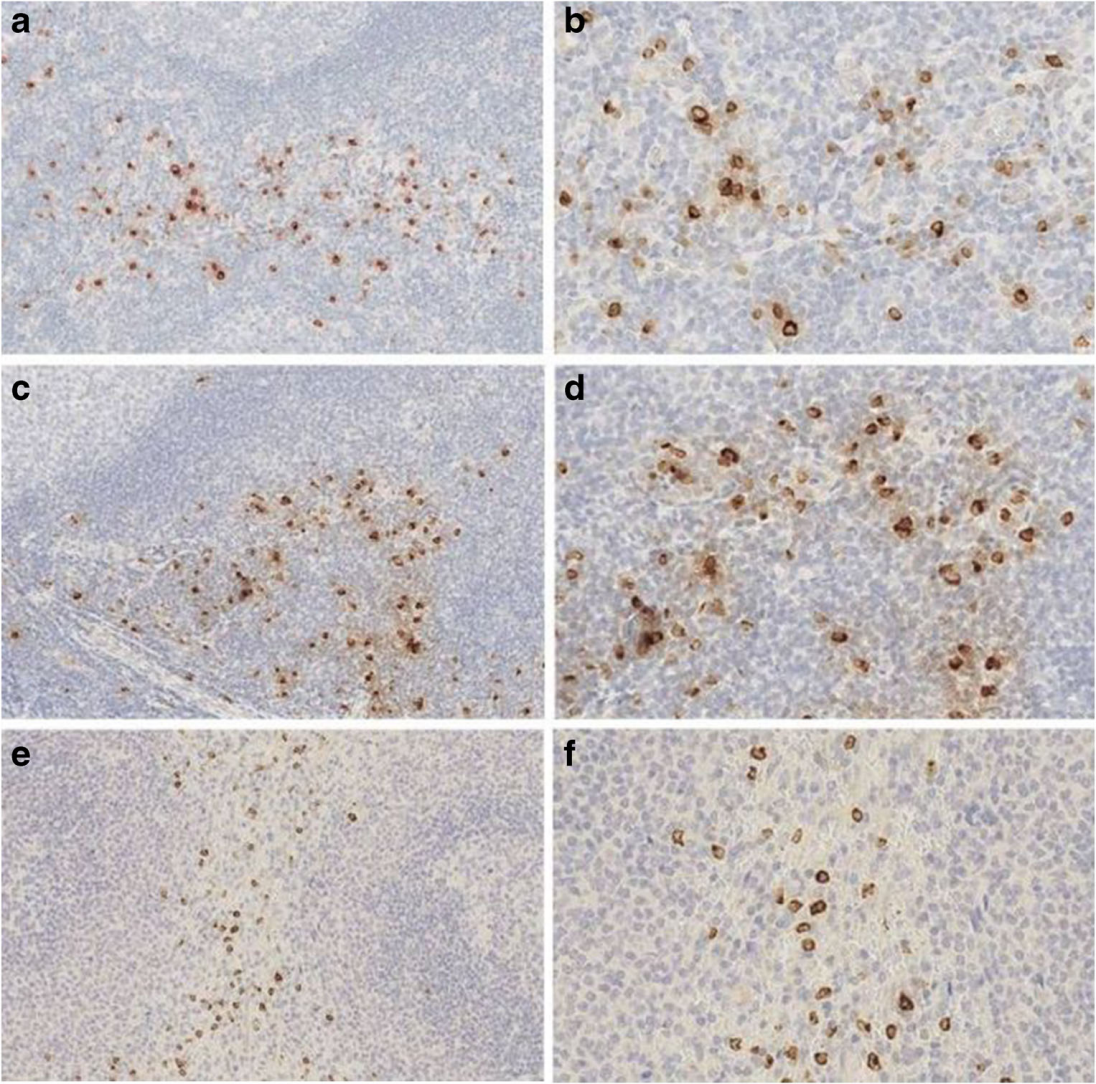
Expression of granulysin in normal lymphoid tissues. Tonsil (**a**, **b**), lymph node (**c**, **d**), spleen (**e**, **f**) [**a**, **c**, **e** original magnification (O.M.): × 10; **b**, **d**, **f** O.M. × 20]

**Fig. 2:**
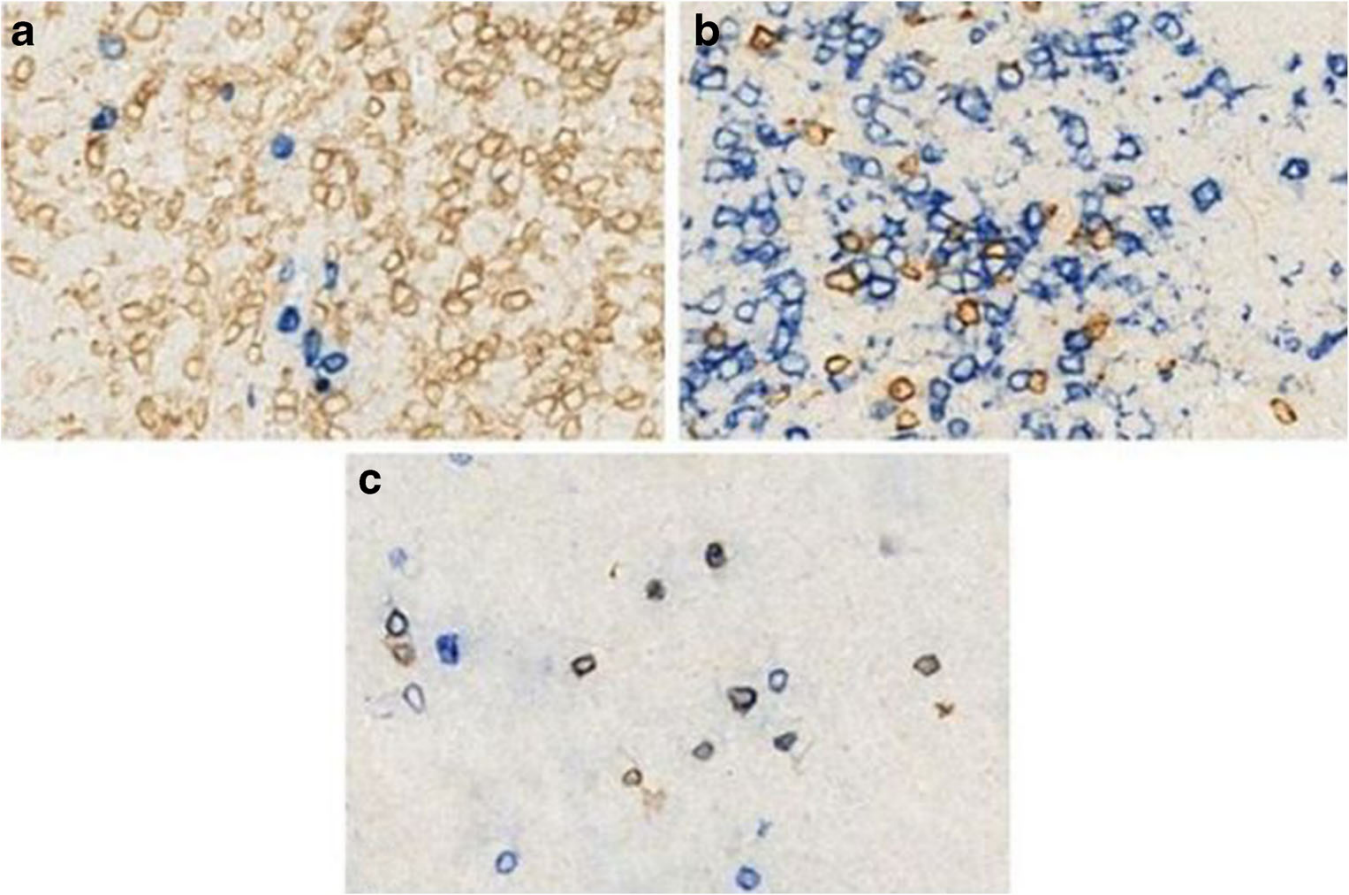
Double immunostaining of granulysin and T/NK cell markers in reactive tonsils. CD4-positive cells are granulysin negative (**a**); a few CD8-positive cells co-expressed granulysin (**b**). The majority of CD56-positive cells were granulysin positive (**c**). In all images, granulysin is in blue with the exception in **b** which is in brown. No counterstaining is performed. [O.M.: **a**– **c** × 20]

Granulysin showed a membranous and intracellular (dot-like) staining restricted to a small population of cells in the interfollicular areas of reactive tonsils and lymph nodes (Fig. 1). In the red pulp of the spleen and in the medulla of the thymus, scattered granulysin-positive cells were seen (Fig. 1). Double immunostaining carried out in tissue sections of reactive tonsil and lymph node demonstrated that the granulysin-positive cells were mostly CD4 negative and few co-expressed CD8. The majority of granulysin-positive cells co-expressed CD56 (Fig. 2).

B cells, plasma cells, macrophages, epithelioid histiocytes and dendritic cells were granulysin negative.

### Granulysin expression in CAEBV

Five lymph nodes with the diagnosis of CAEBV were included in this study. All cases showed EBV infection by EBER-ISH and strongly expressed one or more of the following markers: CD2, CD3, CD8, TIA-1, granzyme B and perforin. Four out of five cases were T cell type CAEBV (also confirmed by PCR detection of a monoclonal TCR rearrangement), and the remaining case was of B cell type.

Only scattered lymphocytes, expressing one or more cytotoxic markers, were granulysin positive.

### Granulysin expression in haematological malignancies

Table 1 summarises the results of granulysin in a series of haematological malignancies.

Granulysin was found to be mainly restricted to some NK/T cell lymphoma subtypes, and the highest expression was observed in ENKTLs (61/86; 71%). All the investigated ENKTL cases were EBER-positive and strongly expressed one or more of the cytotoxic markers (i.e. CD56, granzyme B, TIA-1 and perforin) and/or CD2, CD3, CD7 and CD43 (Table 2).

**Table 2:** Immunohistochemical and in situ hybridization results in nasal-type extranodal NK/T cell lymphomas

Marker	Positivity (*n*/*N*)
Granulysin	71% (61/86)
CD56	98,8% (85/86)
Granzyme B	77% (66/86)
TIA-1	68% (59/86)
Perforin	40% (34/86)
EBER	100% (86/86)
CD2	86% (49/57)
CD3 (cytoplasmic)	76% (65/86)
CD7	75% (6/8)
CD43	100% (42/42)
CD5	3,6% (2/56)
CD30	16% (7/43)

*n* number of cases positive for granulysin, *N* total number of cases, *%* percentage of positive cases

**Fig. 3:**
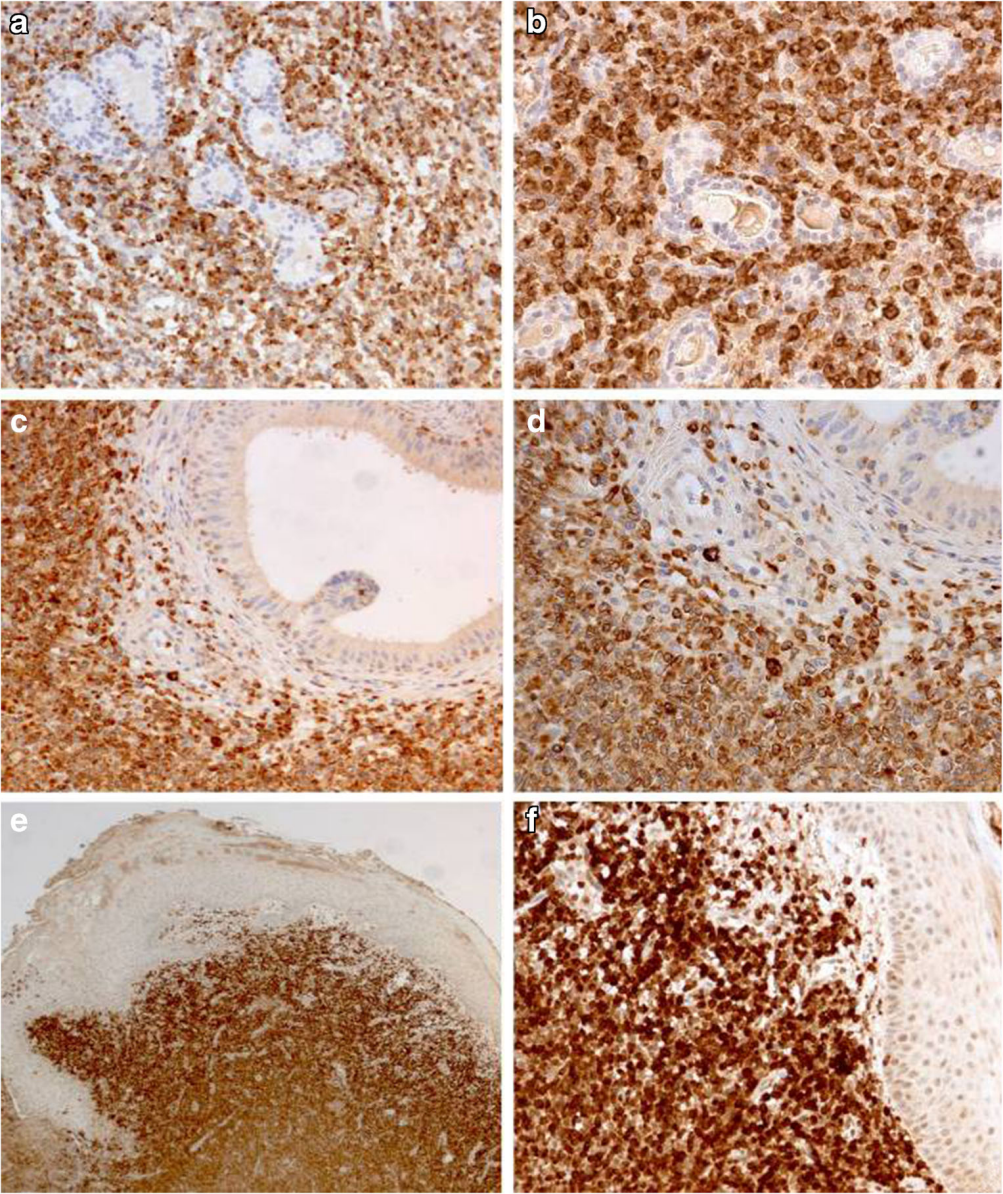
Granulysin ENKTL lymphomas with different organs involvement. Nasal cavity (**a**, **b**), rete testis (**c**, **d**), skin (**e**, **f**) [**a**, **c** O.M. × 10; **e** O.M. × 2.5; **b**, **d**, **f** O.M. × 20]

**Fig. 4:**
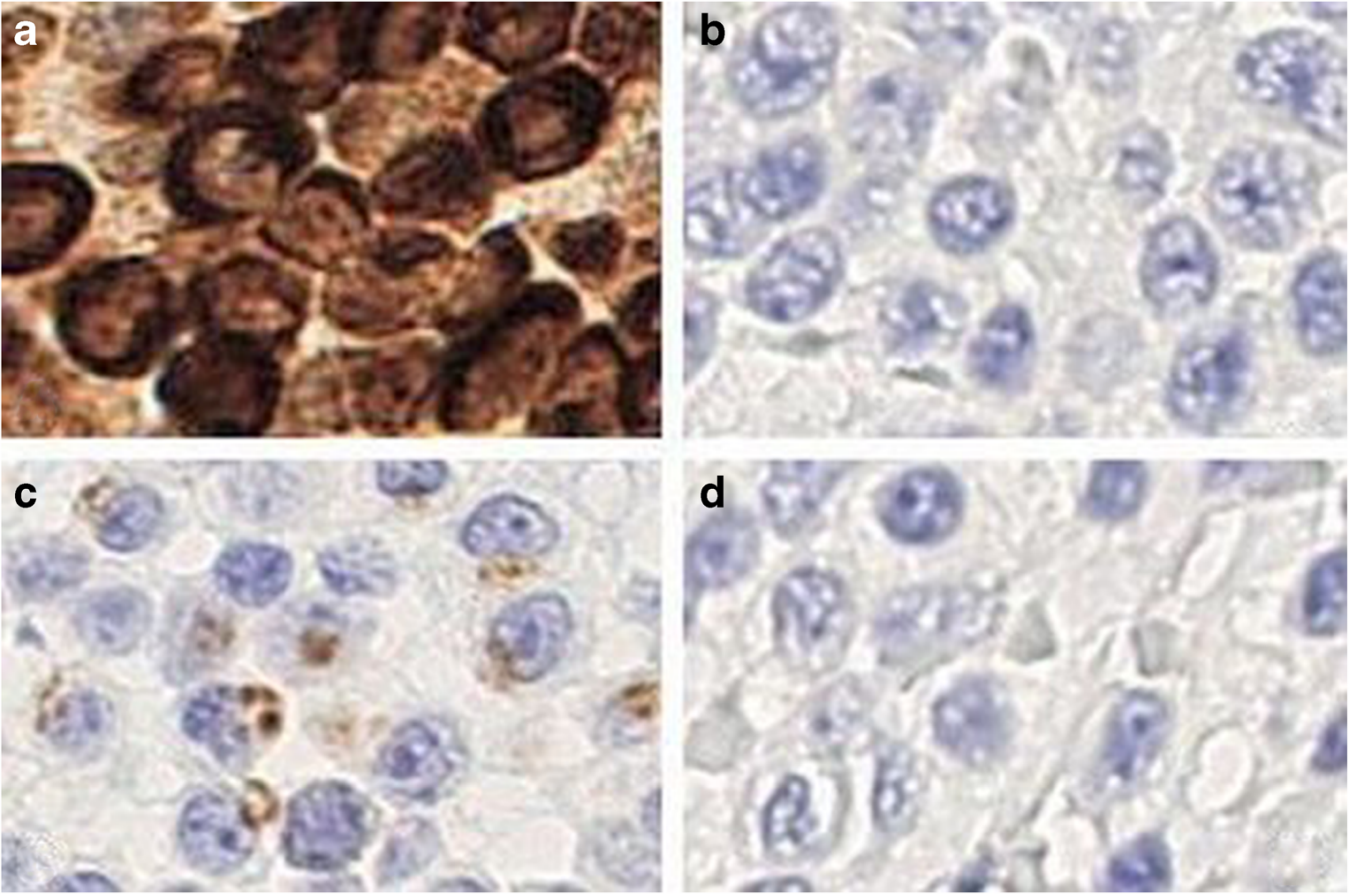
Granulysin is strongly expressed in tumour cells of ENKTL cases that lack other cytotoxic markers. **a** Granulysin, **b** granzyme B, **c** TIA-1, **d** perforin. In the first case, tumour cells are negative for granzyme B and perforin but they express weakly TIA-1. [O.M. **a**–**d** × 40]

**Fig. 5:**
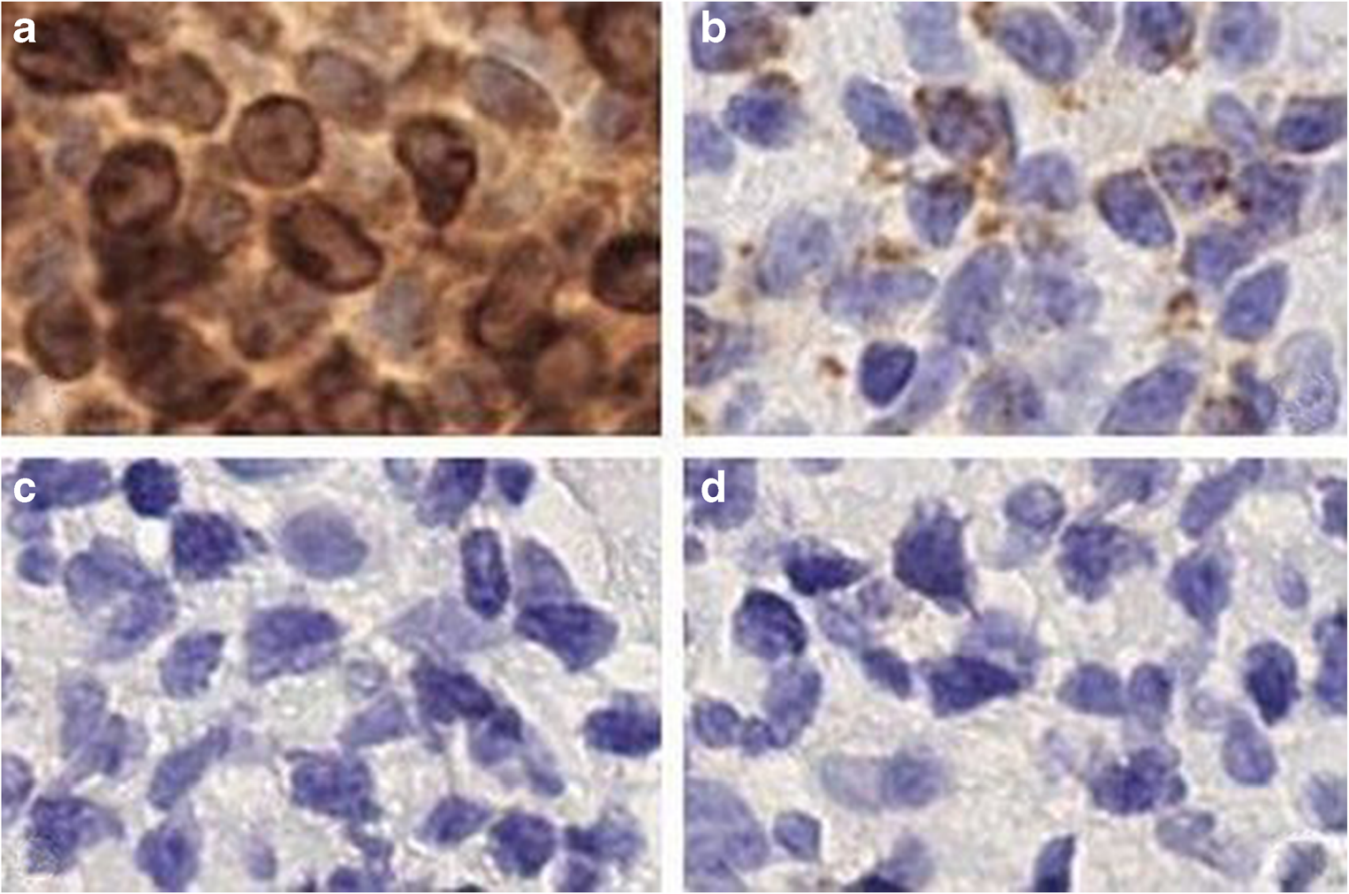
Granulysin is strongly expressed in tumour cells of ENKTL cases that lack other cytotoxic markers. **a** Granulysin, **b** granzyme B, **c** TIA-1, **d** perforin. In the second case, tumour cells are negative for TIA-1 and perforin but they express weakly granzyme B [O.M. **a**–**d** × 40]

The intensity of granulysin in the 61 positive ENKTLs was strong and comparable to that of the other cytotoxic markers, and in each case, more than 75% of tumour cells were positive (score 3, Fig. 3). To note is that 26 out of the 61 granulysin-positive cases showed a statistically significant lower level of expression of the other cytotoxic markers (*p* < 0.01) whereas granulysin was homogeneous and strong in all instances. Table 3 summarises the clinicopathological data of the 26 cases with strong granulysin expression and lack of one or all others cytotoxic markers (Figs. 4 and 5).

**Table 3:** Immunohistochemical features of granulysin-positive ENKTL cases with lower level of expression of the other cytotoxic markers

Case	Site	Age	Sex	CD56	Granzyme B	TIA-1	Perforin	cCD3	CD4	CD8
1	Nasal cavity	22	M	Pos	Neg	Neg	Neg	Pos	Neg	Neg
2	Nasopharynx	54	M	Pos	Neg	Neg	Neg	Pos	Neg	Neg
3	BM	51	M	Pos	Neg	Pos	Neg	Pos	Neg	Neg
4	Stomach	74	M	Pos	Neg	Neg	Pos	Pos	Neg	Neg
5	Maxilla	28	M	Pos	Pos	Neg	Neg	Pos	Neg	Neg
6	Duodenum	56	M	Pos	Pos	Neg	Neg	Neg	Neg	Neg
7	Soft palate	23	F	Pos	Pos	Neg	Neg	Pos	Neg	Pos
8	Nasopharynx	37	M	Pos	Neg	Pos	Neg	Pos	Neg	Neg
9	Nasal septum	17	F	Pos	Neg	Pos	Neg	Pos	Neg	Neg
10	Nasal cavity	50	F	Pos	Pos	Neg	Neg	Pos	Neg	Neg
11	Soft palate	35	F	Pos	Pos	Neg	Neg	Pos	Neg	Neg
12	Nasal septum	8	M	Pos	Pos	Neg	Neg	Pos	Neg	Neg
13	Palate	61	F	Pos	Neg	Pos	Neg	Pos	Neg	Pos
14	Skin/breast	40	F	Pos	Pos	Pos	Neg	Pos	Neg	Neg
15	Stomach	24	M	Pos	Pos	Pos	Neg	Neg	Neg	Neg
16	Nasal fossa	51	M	Pos	Pos	Pos	Neg	Pos	Neg	Neg
17	Nasal septum	38	F	Pos	Pos	Pos	Neg	Pos	Neg	Neg
18	Nasopharynx	42	F	Pos	Pos	Pos	Neg	Pos	Neg	Neg
19	Skin	48	M	Pos	Pos	Pos	Neg	Pos	Neg	Neg
20	Maxilla	60	M	Pos	Pos	Pos	Neg	Pos	Neg	Neg
21	Caecum	26	F	Pos	Pos	Neg	Pos	Pos	Neg	Neg
22	Testicle	18	M	Pos	Pos	Pos	Neg	Pos	Neg	Neg
23	Testis	51	M	Pos	Neg	Neg	Neg	Pos	Neg	Neg
24	Skin	24	M	Pos	Pos	Neg	Pos	Neg	Neg	Neg
25	Nasal fossa	44	F	Pos	Pos	Pos	Neg	Pos	Neg	Neg
26	Large bowel	80	M	Pos	Neg	Pos	Neg	Pos	Neg	Neg

All cases were granulysin and EBER positive. TCR rearrangement is not available in all cases but not two (*n* 3 and 24) that are polyclonal

*Pos* positive, *Neg* negative, *M* male, *F* female, *BM* bone marrow, *cCD3* cytoplasmic CD3

In the remaining 25 ENKTL cases (29%), the neoplastic cells were consistently granulysin negative; the clinicopathological data of these cases are detailed in Table 4. We may conclude that the majority of granulysin-negative ENKTCL cases lack expression of perforin. Moreover, all granulysin-negative cases arose from upper digestive tract (nasal cavity, nasopharynx, paranasal sinus and palate) while many strongly granulysin-positive cases are from skin, testis and gastrointestinal tract (stomach, small intestine and large bowel).

**Table 4:** Immunohistochemical features of granulysin-negative ENKTL cases

Case	Site	Age	Sex	CD56	Granzyme B	TIA-1	Perforin	cCD3	CD4	CD8
1	Nasal cavity	41	M	Pos	Pos	Pos	Neg	Pos	Neg	Neg
2	Palate	65	F	Pos	Neg	Pos	Neg	Pos	Neg	Neg
3	Palate	47	M	Pos	Pos	Neg	Neg	Pos	Neg	Pos (weak)
4	Nasal fossa	53	M	Pos	Pos	Pos	Neg	Pos	Neg	Neg
5	Nasal fossa	58	F	Pos	Neg	Pos	Neg	Pos	Neg	Neg
6	Nasal fossa	54	M	Pos	Pos	Neg	Neg	Pos	Neg	Neg
7	Nasal sinus	45	M	Pos	Pos	Pos	Neg	Pos	Neg	Neg
8	Nasal sinus	44	F	Pos	Pos	Pos	Neg	Pos	Neg	Neg
9	Lymph node	51	F	Pos	Pos	Pos	Pos	Pos	Neg	Neg
10	BM	52	M	Pos	Pos	Pos	Pos	Pos	Neg	Neg
11	Palate	35	M	Pos	Neg	Pos	Neg	Pos	Neg	Neg
12	Nasal cavity	42	M	Pos	Pos	Pos	Neg	Pos	Neg	Neg
13	Nasal cavity	61	F	Pos	Neg	Pos	Pos	Neg	Neg	Neg
14	Nasal cavity	54	M	Pos	Pos	Pos	Neg	Pos	Neg	Neg
15	Oral cavity	63	F	Pos	Pos	Pos	Pos	Pos	Neg	Neg
16	Palate	47	M	Pos	Pos	Pos	Neg	Pos	Neg	Neg
17	Palate	52	M	Pos	Pos	Neg	Neg	Pos	Neg	Neg
18	Nasal cavity	48	F	Pos	Pos	Neg	Neg	Pos	Neg	Neg
19	Maxilla	41	M	Pos	Pos	Pos	Neg	Pos	Neg	Neg
20	Lymph node	60	M	Pos	Pos	Pos	Neg	Pos	Neg	Neg
21	Nasal cavity	53	M	Pos	Pos	Pos	Pos	Pos	Neg	Neg
22	Nasal fossa	49	F	Pos	Pos	NA	NA	Pos	Neg	Neg
23	Nasal cavity	41	M	Pos	Pos	NA	NA	Pos	Pos (focal)	Neg
24	Nasal cavity	57	F	Pos	Pos	Pos	NA	Pos	Neg	Pos
25	Nasal cavity	54	M	Pos	Pos	Pos	NA	Pos	Neg	Pos

All cases were EBER positive and granulysin negative. TCR rearrangement is not available in all cases but not one (*n* 11) that is polyclonal

*Pos* positive, *Neg* negative, *NA* not available, *M* male, *F* female, *BM* bone marrow

Furthermore, weak granulysin positivity (score 1) was seen in 4 out of 15 (26%) ALK-negative anaplastic large cell lymphomas (ALK-ALCL), 1 out of 8 (12%) enteropathy-associated T cell lymphomas (EATL, all cases are type I) and 2 out of 50 (4%) peripheral T cell lymphomas, NOS (PTCL, NOS), both showing gamma/delta genotype.

All cases of non-Hodgkin B cell lymphoma, Hodgkin lymphoma and plasma cell myeloma were granulysin negative.

## Discussion

Expression of granulysin in normal and neoplastic lymphoid tissues has been only rarely characterised [8]. In the current study, we show that granulysin is expressed in cytotoxic/NK lymphocytes and in subsets of NK/T cell derived lymphomas.

In normal tissues, granulysin marked small numbers of T/NK-cells within the interfollicular areas of tonsils, lymph nodes and in the red pulp of the spleen. These findings correlate with a previous report describing the expression of granulysin in cytotoxic NK/T cells [4].

Our findings showed that the majority of granulysin-positive cells co-expressed CD56 while most of the CD4+ and CD8+ T cells were negative for granulysin. This suggests that the expression of granulysin is mainly restricted to NK cells and derived neoplasms. Accordingly, we found that the highest level (score 3) of granulysin expression was in a subset of ENKTLs (71%) and higher when compared to that of other cytotoxic markers (*p* < 0.01). This finding supported the results of a previous investigation that reported elevated serum levels of granulysin in patients with NK/T cell lymphoma [8]. In the present study, we are not able to quantify serum levels of granulysin from blood samples and to correlate with its expression in tumour tissue. The importance of this analysis was pointed out in a previous investigation being an indicator of disease burden. A significant higher serum level found in ANKL than in ENKTL correlated with increased cancer cells load in the NK cell leukaemia [8]. In fact, serum levels of granulysin significantly decreased after treatment, suggesting its potential role as prognostic marker [8].

The staining pattern of granulysin was comparable to that of commonly used cytotoxic markers granzyme B, TIA-1 and perforin, thus suggesting its diagnostic usefulness in the clinical setting of ENKTLs. This was further highlighted by strong granulysin positivity observed in a proportion of ENKTLs (30%) that lacked expression of one or more of the classical cytotoxic markers.

Our results showing granulysin expression in a small proportion of other lymphomas with cytotoxic phenotypes, i.e. ALK-negative ALCL, EATL and PTCL NOS support previous observations of granulysin positivity in systemic ALCL of childhood, in mycosis fungoides (MF) and Sézary syndrome [21, 22]. However, both studies did not give details regarding the number of investigated cases and incidence of granulysin positivity or staining pattern characteristics. In our analysis, we found that in adult ALK-negative ALCLs, the expression of granulysin was less strong and intense than in ENKTL cases and in addition restricted to a small proportion of tumour cells (less than 25%; score 1).

Although we found granulysin expression in the majority of ENKTCL cases, further experiments with NK and cytotoxic T cell lines are required to demonstrate granulysin as a cytotoxic marker of NK cells and derived lymphomas and to correlate with cell of origin (NK versus T cell).

Finally, it should be mentioned that granulysin may represent a potential therapeutic target for NK/T cell lymphomas that are characterised by a poor prognosis [5]. Notoriously, successful disease control is rarely achieved in most ENKTL patients with relapsed or refractory diseases, and consistent with several international clinicopathological studies [23], the treatment of ENKTL nasal type differs according to the stage of the disease. Patients with systemic disease tend to be treated with L-asparaginase-containing regimens and although showed best results, toxicity is not negligeable [23]. Thus, development of tailored drugs targeted to novel biomarkers, and granulysin might represent one, should be considered in order to improve treatment outcome.

In conclusion, our study suggests granulysin as an additional molecule for cytotoxic and NK/T cells as well as a reliable diagnostic marker for NK/T cell-derived lymphoma subtypes and useful for those ENKTLs that lack expression of one or more of the commonly used cytotoxic markers.

Our results open new insights for future investigations aimed to validate the clinical value of granulysin to monitor disease in biopsies post-therapy.
